# Brain Network Response to Acupuncture Stimuli in Experimental Acute Low Back Pain: An fMRI Study

**DOI:** 10.1155/2015/210120

**Published:** 2015-06-16

**Authors:** Yu Shi, Ziping Liu, Shanshan Zhang, Qiang Li, Shigui Guo, Jiangming Yang, Wen Wu

**Affiliations:** ^1^Department of Rehabilitation, Zhujiang Hospital, Southern Medical University, Guangzhou 510282, China; ^2^Department of General Surgery, Zhujiang Hospital, Southern Medical University, Guangzhou 510282, China; ^3^Department of Radiology, Zhujiang Hospital, Southern Medical University, Guangzhou 510282, China

## Abstract

Most neuroimaging studies have demonstrated that acupuncture can significantly modulate brain activation patterns in healthy subjects, while only a few studies have examined clinical pain. In the current study, we combined an experimental acute low back pain (ALBP) model and functional magnetic resonance imaging (fMRI) to explore the neural mechanisms of acupuncture analgesia. All ALBP subjects first underwent two resting state fMRI scans at baseline and during a painful episode and then underwent two additional fMRI scans, once during acupuncture stimulation (ACUP) and once during tactile stimulation (SHAM) pseudorandomly, at the BL40 acupoint. Our results showed that, compared with the baseline, the pain state had higher regional homogeneity (ReHo) values in the pain matrix, limbic system, and default mode network (DMN) and lower ReHo values in frontal gyrus and temporal gyrus; compared with the OFF status, ACUP yielded broad deactivation in subjects, including nearly all of the limbic system, pain status, and DMN, and also evoked numerous activations in the attentional and somatosensory systems; compared with SHAM, we found that ACUP induced more deactivations and fewer activations in the subjects. Multiple brain networks play crucial roles in acupuncture analgesia, suggesting that ACUP exceeds a somatosensory-guided mind-body therapy for ALBP.

## 1. Introduction

Low back pain (LBP) is one of the most common clinical syndromes and affects 80–85% of people at some point in their life. This disorder typically causes serious socioeconomic problems, including health and economic issues, and even medications abuse [1, 2]. Most LBP does not have a definitive cause, and it has been shown that traditional Chinese medical acupuncture has beneficial effects on this intractable pain [3, 4].

Previous brain imaging studies have found that external stimuli, including acute experimental pain, could evoke deactivations in the default mode network (DMN), a network believed to be involved in the areas of memory and social affective and self-referential cognition [5, 6]. Pain stimulus could also induce extensive activations in the limbic system [anterior cingulated cortex (ACC), periaqueductal gray (PAG), prefrontal cortex] and somatosensory system (thalamus, primary somatosensory cortex (S1), secondary somatosensory cortex (S2), posterior parietal cortices, insula, supplementary motor area, striatum, and cerebellum) areas [7, 8], as well as the pain matrix (S1, S2, insular, frontal lobe and parietal lobe). The pain matrix showed a strong relationship with pain, which plays an important role in the conduction and communication of pain [9]. Moreover, research on acupuncture analgesia has recently become increasingly popular. Some researchers have found that acupuncture yields activations in the attentional- and emotional-related regions (DMN, dorsomedial, and dorsolateral prefrontal cortex (dmPFC and dlPFC)), and deactivations in the somatosensory system (left anterior insula, bilateral S1, and S2) compared with cutaneous stimuli. Therefore, some researchers considered that acupuncture could function as a somatosensory-guided mind-body therapy [10], while others held the belief that the effect of acupuncture may not be limited to DMN or the somatosensory system. These researchers found that acupuncture evoked more deactivations of the limbic-paralimbic-neocortical network, which was thought to be centered on the limbic system, but fewer activations in the somatosensory and attentional systems compared with cutaneous stimuli [11]. Taken together, these fMRI studies, with or without experimental heat pain on limbs, have greatly contributed to our understanding of the analgesic mechanism of acupuncture; however, only a few have examined clinical pain, with the least focus on acute low back pain (ALBP). Hence, it is interesting to explore how acupuncture modulates the brain networks in ALBP subjects using fMRI.

There are two major barriers preventing fMRI studies on clinical ALBP. Firstly, it is hard to distinguish ALBP qualitatively and quantitatively from the multiple potential etiologies and their various degrees. Moreover, it is difficult to conduct experiments because ALBP is characterized by sudden onset and aggravation. In addition, using experimental heat pain to simulate ALBP is problematic, because it is difficult to expose the volunteers' back when they are lying down in the MRI scanner.

In this block design fMRI study, we introduced a simple and quantitative ALBP model induced by hypertonic saline injection in healthy volunteers to investigate the neural mechanism of acupuncture analgesia. For each experimental ALBP subject, we delivered comparable therapeutic stimuli, including ACUP and SHAM at the BL40 acupoint (Weizhong) on the right lower limb. Moreover, before and after the application of therapeutic stimuli, we collected data on subjective pretreatment and posttreatment LBP and their corresponding acupuncture sensations.

Therefore, experimental ALBP could not only act as a clinical LBP but also as evaluation criteria for the therapeutic stimuli.

## 2. Materials and Methods

### 2.1. Subjects and Acupoint

Twenty-eight healthy, right-handed subjects (11 women; age range: 22–30 years) participated in this study. All participants had some knowledge of acupuncture due to previous cultural exposure; had never received acupuncture treatment; had a body mass index within the standard range (±10%); had no psychiatric or medical illnesses (i.e., multiple sclerosis and epilepsy); and had no painful episodes (including dysmenorrhea) or did not take any drugs (i.e., antipyretics and sleeping pills) within the last month. The study was conducted with the understanding and written consent of each subject. All experiments and protocols were approved by the Ethics Committee of Zhujiang Hospital Affiliated to Southern Medical University, China.

**Figure 1:**
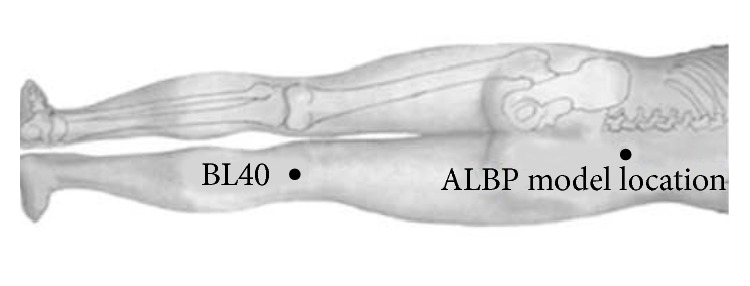
The location of the BL40 and ALBP model.

**Figure 2:**
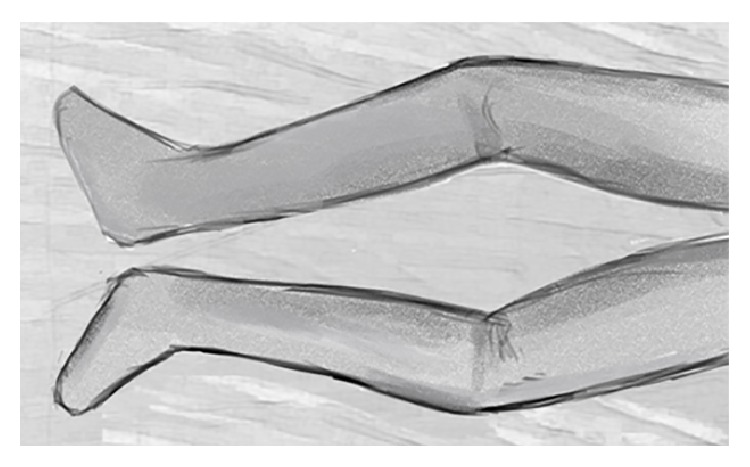
The posture of the subjects when inserting the needle at the point.

In traditional Chinese medicine, BL40 (Weizhong acupoint) is considered as one of the four most important acupoints and proven to have unique efficacy in the treatment of ALBP. For instance, A Complete Collection of Acupuncture and Moxibustion, written by Xufeng who is an acupuncture-moxibustion expert in the Ming Dynasty, states that lumbar-back problems could be treated by puncturing this acupoint [12]. BL40 is anatomically located at the midpoint of the transverse crease of the popliteal fossa (Figure 1); therefore, each subject's keen was leaned on mattress to keep lower limbs in a valgus position for therapeutic stimuli (Figure 2).

### 2.2. Experimental Procedures

Anatomical scans of the brain and functional images of sensory control stimulation were collected prior to stimulation imaging. Initially, the subjects were subjected to a baseline (the normal time) resting state (rs) MRI scan for 6 min. A preliminary ALBP model was induced in the right lower back muscle of each subject using a method modified from previous studies [13, 14]. In the experimental ALBP model, we could control the levels of pain, which gave the subjects a similar level of pain in the experiment, resulting in a smaller margin of error. The variety of clinical LBP cannot meet this requirement.

**Figure 3:**
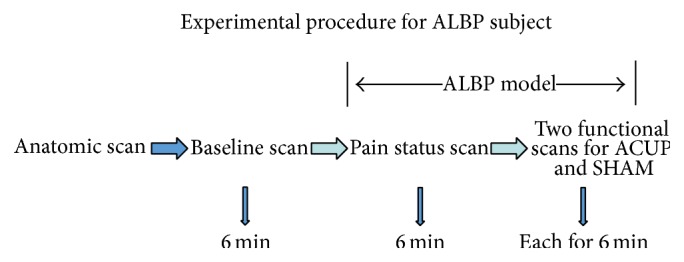
The experimental paradigm for the ALBP subjects included five steps.

**Figure 4:**
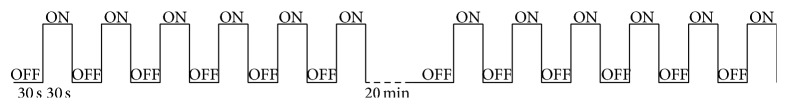
Each functional scan lasted for 6 min, including six OFF-ON blocks; the time interval between the two functional scans was 20 min. During the six ON blocks of each functional scan, ACUP or SHAM was applied at BL40.

After the baseline scan, we located an injection point 2 cm lateral to the spinous process of the fourth lumbar vertebra for the ALBP model. Thereafter, we filled an in-dwelling needle (24 gauge) with sterile hypertonic saline (10 mL, 5%) and attached it via a long connecting tube to a computer-controlled power injector (Spectris Solaris EP; Medrad, Inc., Warrendale, PA, USA), before vertically inserting it into the above-described location at a depth of 1.5 cm (Figures 1 and 3). After 1 min, the hypertonic saline was injected intramuscularly from the above-mentioned computer-controlled power injector into the ALBP subject. This injection included a bolus injection (0.1 mL within 5 s) and subsequent continuous injection (0.15 mL/min) to produce persistent ALBP. During the first 6 min of ALBP stimulation, we performed an rs-fMRI scan to evaluate the pain status. After the pain rsfMRI scan, we obtained two functional scans for each ALBP subject: one scan during acupuncture stimulation (ACUP) and one scan during tactile stimulation (SHAM) pseudorandomly, with ALBP occurring continually throughout the scanning process. The ACUP or SHAM run comprised a block design with six 30 s blocks of rest time (OFF block) interspersed between six 30 s blocks of stimulation (ON block); ACUP (or SHAM) was administered at BL40 by the same experienced acupuncturist during the six ON blocks of each functional scan. Each functional scan lasted for 6 min, and the time interval between the two functional scans (ACUP and SHAM) was set at 20 min to maximize washout of the sustained effects induced by the former therapeutic stimulation (Figure 4). All MRI scans were performed with each subject laying still in a Philips 3.0 T Achieva scanner (Royal Philips Electronics, Eindhoven, Netherlands) with their eyes and ears covered.

Notably, we had examined the effects of different injection speeds (0.1 mL/min, 0.15 mL/min, 0.2 mL/min) of the hypertonic saline after a bolus injection (0.1 mL within 5 s), in the preliminary experiment. We found that 0.15 mL/min was most suitable one for our experiment, because it induced a persistent moderate-high pain in ALBP subject.

### 2.3. Acupuncture Modulation

ACUP was administered by inserting a nonmagnetic (pure silver), 0.4 mm-diameter, 60 mm-long acupuncture needle (Beijing Zhongyan Taihe Medicine Co., Ltd, Beijing, China) vertically into BL40 at a depth of approximately 2 cm (Figure 1). To obtain a subjective acupuncture sensation, namely,* de qi* sensation [15], the needle was manually twirled (±180°) at 1 Hz with “even reinforcing and reducing” needle manipulation in traditional Chinese medicine, while SHAM was delivered with a von Frey monofilament. The acupuncturist poked this monofilament through a needle-guide tube and tapped it gently over the skin of the BL40 with the same amplitude and rate as that used during ACUP [16].

Considering that SHAM may cause subjective bias towards the stimulation, all subjects were asked to keep their eyes and ears closed in order to prevent them from discriminating the therapeutic stimulation. Moreover, all subjects were purposely misguided that they would receive two different forms of acupuncture and needed to concentrate on the degree of acupuncture sensations of BL40. Therefore, SHAM aimed to control for not only the superficial and cutaneous somatosensory effects around BL40 but also the cognitive processing induced by the subject's expectation of “ACUP” [17].

### 2.4. Psychophysical Data Collection and Analysis

After each MRI scan, each subject was asked to quantify the* de qi* sensations at BL40 using a 10-point scale (0 = none, 1–3 = mild, 4–6 = moderate, 7–9 = strong, and 10 = unbearable) [16]. Moreover, each ALBP subject was asked to rate the intensity of LBP before and after each MRI scan using a 10-point visual analog scale (0 = none, 1–3 = mild, 4–6 = moderate, 7–9 = strong, and 10 = unbearable). Correspondingly, the scores of the* de qi* sensations were compared between ACUP and SHAM in the ALBP group [18] and pre- and posttreatment LBP between ACUP and SHAM in the ALBP group, using the Wilcoxon signed-rank test; *P* values < 0.05 were considered to be statistically significant (SPSS 13.0, IBM Corporation, NY, USA).

### 2.5. Imaging Data Collection and Analysis

Structural and functional scans were acquired with a 3.0 T Philips Achieva MRI System (Royal Philips Electronics, Eindhoven, Netherlands) with an 8-channel head array coil equipped for echo planar imaging. The images were axial and parallel to the anterior commissure-posterior commissure line, which covered the whole brain. Structural images were collected prior to functional imaging using a T1-weighted fast spin echo sequence (repetition time/echo time = 500/14 ms, flip angle = 90°, 0.859 mm × 0.859 mm in-plane resolution, slice thickness = 1 mm). Blood oxygenation level-dependent functional imaging was acquired using a T2^*∗*^-weighted, single-shot, gradient-recalled echo planar imaging sequence (repetition time/echo time = 2000/40 ms, flip angle = 90°, 3.4 mm × 3.4 mm in-plane resolution, 180 time points for a total of 360 seconds). In addition, fMRI image collection was preceded by 5 dummy scans to minimize gradient distortion.

#### 2.5.1. Preprocessing of Experimental MRI Data

Data analysis was performed with SPM8 software (http://www.fil.ion.ucl.ac.uk/spm/). Preprocessing includes motion correction, slice-timing correction, normalization to the Montreal Neurological Institute standard brain (MNI152), and spatial smoothing with a Gaussian kernel of full width at half maximum of 8 mm. For motion correction, the subject's data was excluded if translation or rotation of the subject's head movements was more than 1.5 mm or 1.5°.

*(1) Rs-fMRI Data Analysis*. The preprocessing data were then processed to produce regional homogeneity (ReHo) map image files. The ReHo analysis was performed according to previous reports [18] and calculated using Kendall's coefficient to measure ReHo or the similarity of a ranked time series from a given voxel with that of its nearest 26 neighboring voxels in a voxelwise manner. Kendall's coefficient value was calculated for this voxel, and an individual Kendall's coefficient map was obtained for each subject. Each ReHo map was divided by its own mean ReHo within the mask for standardization purposes [18]. The ReHo value differences between the pain status and baseline were calculated using two-tailed, paired *t*-tests (*P* < 0.05) and corrected for multiple comparisons false discovery rate (FDR). The results were displayed using BrainNet viewer software (http://www.nitrc.org/projects/bnv/).

*(2) Task fMRI Data Analysis.* In the first-level analysis, the preprocessing task functional data were modeled using a general linear model. Explanatory variables, including the stimulation task (ACUP or SHAM, ON status) and the OFF status, were modeled using a boxcar function that convolved with the canonical hemodynamic response function in SPM8. Subsequently, parameter estimates were assessed using least-square regression analyses. Next, statistical parametric maps of the stimulation task (ACUP or SHAM) minus the OFF status contrast were collected at each voxel for each subject. In the second-level analysis, a one-sample *t*-test was applied to ACUP (or SHAM) minus the OFF status to assess the main effect of the stimulation, and a paired *t*-test was applied to ACUP minus SHAM to assess differences between the ACUP and SHAM conditions in the ALBP subjects. The threshold was set (*P* < 0.05) and corrected for multiple comparisons (FDR: <0.05). The resulting images were displayed using rest software (http://restfmri.net/forum/rest).

## 3. Result

### 3.1. Psychophysical Responses

**Figure 5:**
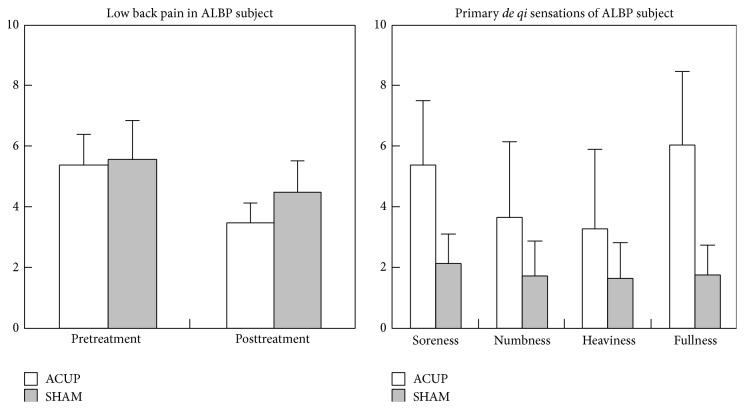
Results of psychophysical analysis in ACUP and SHAM. For ALBP subjects, there were significant differences between the ACUP and SHAM in the mean value of posttreatment pain (*P* = 0.043), soreness (*P* = 0.014), and fullness (*P* = 0.001).

The intensity of the lower back pain and* de qi* sensations are expressed below as mean ± standard deviation. Soreness, numbness, fullness, and heaviness were the primary* de qi* sensations in the current study. In the ALBP group, the mean values of pretreatment LBP were 5.40 (S.D. = 0.98) and 5.60 (S.D. = 1.24) and those of posttreatment LBP were 3.47 (S.D. = 0.75) and 4.51 (S.D. = 1.06) for ACUP and SHAM, respectively. There were significant differences in the score for the soreness and fullness between ACUP and SHAM for ALBP subjects (Figure 5).

### 3.2. fMRI Results

**Figure 6:**
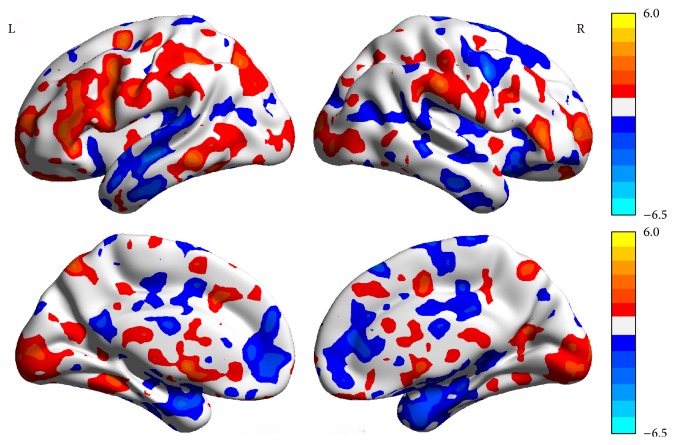
The brain network change in the pain status (pain status and baseline paired *t*-test).

Compared with baseline (the normal time), the pain status showed higher ReHo values in the right medial prefrontal cortex (mPFC), right middle frontal gyrus, right insula, right precuneus (PCN), right parahippocampus (PHP), and right posterior lobe-cerebellar tonsil. However, the pain status showed lower ReHo values in the right superior temporal gyrus, left middle temporal gyrus, left S1, left ACC, left PHP, and right inferior parietal lobule (*P* < 0.05, FDR < 0.05, Table 1, Figure 6).

**Figure 7:**
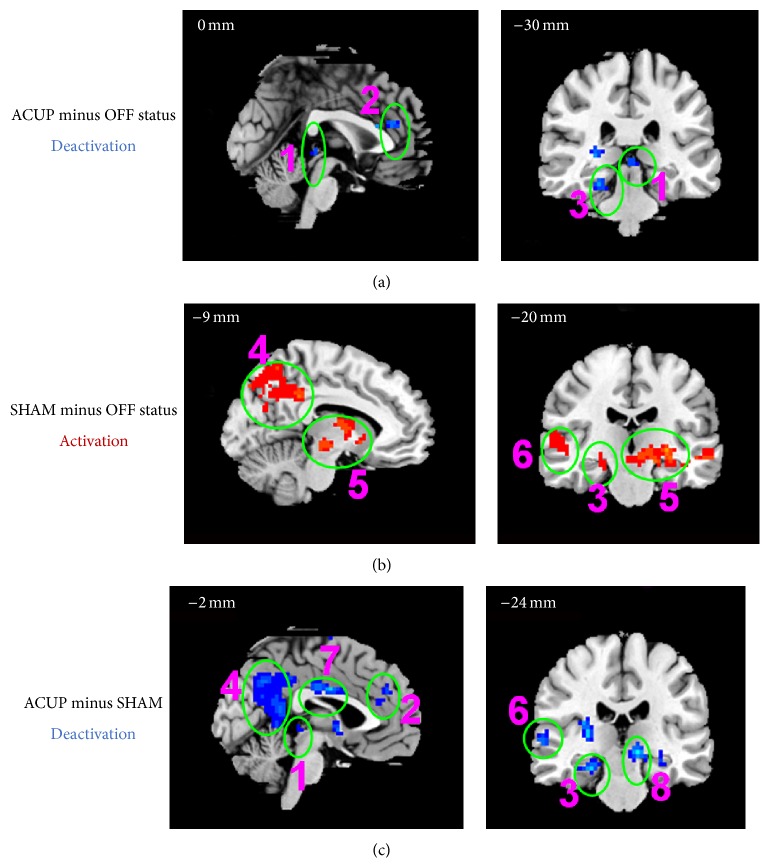
The fMRI signal increases and decreases in cortical and subcortical brain structures, (1) PAG; (2) pACC, aMCC, and anterior dmPFC; (3) PHP and HP; (4) PCN, PCC, and RSC; (5) striatum, thalamus, red nucleus, and substantia nigra; (6) lateral temporal cortex; (7) pMCC; (8) mammillary body.

**Figure 8:**
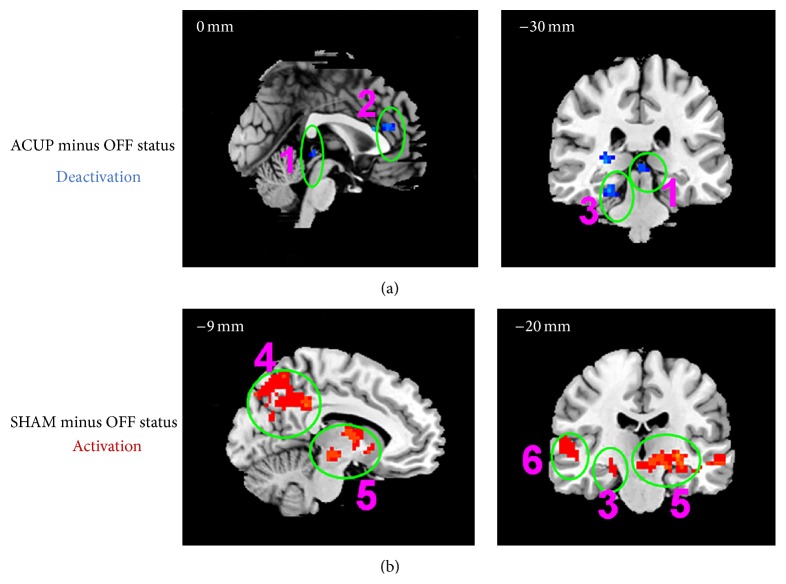
The fMRI signal increases evoked by ACUP and SHAM, (1) right insula and frontal operculum cortex; (2) dlPFC; (3) supramarginal gyrus/angular gyrus; (4) orbitofrontal cortex; (5) lateral temporal cortex and temporal pole.

Compared with the OFF status, ACUP significantly affected the activations and deactivations; deactivations were found in the somatosensory system (left primary motor cortex (M1), S2, and frontal eye field), limbic system (left insula and mammillary body, right hippocampus (HP), bilateral dmPFC, pregenual ACC (pACC), PAG, and PHP), pain matrix (left S1, left insular, temporal lobe, and frontal lobe), DMN (right angular gyrus, supramarginal gyrus, lateral temporal cortex, HP, bilateral dmPFC, and PHP), and bilateral thalamus. The activations, including the right M1, S1, and bilateral supplementary motor areas, right insula, and pMCC, were limited (*P* < 0.05, FDR < 0.05, Table 2, Figures 7 and 8).

Compared with the OFF status, SHAM only produced limited deactivations, such as those in the left insula, left frontal operculum, and left M1, while widespread activations included the somatosensory system (right frontal eye field and bilateral supplementary motor area), attentional system (bilateral dlPFC), limbic system (right frontopolar area, bilateral orbitofrontal cortex, PCN, PHP, HP, temporal pole, amygdala, mammillary body, and PAG), DMN (bilateral angular gyrus, supramarginal gyrus, PCN, PHP, HP, and temporal pole), bilateral thalamus, cerebellum anterior lobe, and lateral occipital gyrus (*P* < 0.05, FDR < 0.05, Table 3, Figures 7 and 8).

Compared with SHAM, ACUP only produced limited activations, including those in the right insula and right M1. In contrast, widespread deactivations were observed, including those in the somatosensory system (left supplementary motor area, bilateral frontal eye field), attentional system (right dlPFC), limbic system (left PAG, bilateral pACC, dmPFC, PHP, HP, and mammillary body), DMN (right supramarginal gyrus, angular gyrus, bilateral dmPFC, PHP, and HP), bilateral thalamus, cerebellar anterior lobe, and lateral occipital gyrus (*P* < 0.05, FDR < 0.05, Table 4, Figures 7 and 8).

## 4. Discussion

To the best of our knowledge, this is the first fMRI study to investigate how acupuncture modulates the brain networks in experimental ALBP subjects. Behaviorally, we delivered similar pain to every subject in accordance with the ALBP model, whereas, compared with SHAM, ACUP showed stronger acupuncture sensations and weaker pain sensations, suggesting that acupuncture alleviated ALBP. As previously found, our fMRI analysis showed that ACUP induced more deactivations but less activations compared with pain status and SHAM. Furthermore, these deactivations in the ALBP subjects were mostly in the regions of the limbic system and DMN, including the antinociceptive and affective (pACC, PAG, aMCC, mammillary body, and dmPFC) and memory (DMN and mammillary body) related brain regions [16]. In contrast, the activations in the ALBP subjects were found in the attentional (dmPFC, dlPFC, pMCC, and right insula) and somatosensory system (right S1, M1, and insula) related regions compared with baseline [10]. Therefore, our results showed that multiple brain networks play important roles in modulating ALBP.

### 4.1. The Network Change in the Pain Status

Similar to other pain stimulation research, the results indicated higher ReHo values in some areas of the brain network. The right mPFC, right middle frontal gyrus, right insula, and right PCN are included in the pain matrix, which has a strong relationship with pain. Different parts of the matrix play different roles in the generation and transmission of pain; for example, S1 and S2 are associated with algesthesia, while the insular cortex and anterior cingulate are associated with the emotional component of pain [9]. The higher ReHo values in the pain matrix represented the pain state via the ALBP model. The mPFC is associated with the processing of emotional information and mediates the functional interactions among the brain regions that participate in pain processing [19, 20], whereas PCN is likely involved in the shifting of attention between different spatial locations [21]. Therefore, changes in ReHo may reflect pain accompanied by the processing of emotionally intense information.

ACC participates in pain perception and integration of the sensory, attentional, and cognitive components of pain [22, 23]. The decrease of ReHo in ACC suggests a reduction in efficient pain processing or compensatory damage in functionally relevant regions such as the prefrontal cortex and caudate [24]. Pain is well documented to potentially interrupt cognition and sustained attention to a direct action toward a painful stimulus or threat [25]. The insula is an important component of the pain system, and its functions involve judgment about potential dangers [26]. The results showed higher ReHo values in the right insula, possibly indicating an increase in the judgment function and evasive actions during the pain state; because the insula also participates in learning and memory regarding pain [27], the higher ReHo values in the insula indicate increased function. The ACC and insula exhibited higher ReHo values in the pain matrix during experimental LBP. The negative correlations between ACC and the insula were enhanced, suggesting that the anterior insula reduces the response to peripheral nociceptive stimuli via a self-control function.

Furthermore, the brain regions with decreased ReHo values were concentrated in the left hemisphere, which verifies Naqvi's conclusion that this hemisphere corresponds to the affective consequences of pain, whereas the right hemisphere corresponds mainly to homeostatic and autonomic control [28].

### 4.2. The Effect of Acupuncture in the Brain Network

#### 4.2.1. Limbic System

Interest in the role of PAG and ACC for pain modulation has a long history [7, 16, 29–32]. Anatomically, nociceptive signals can ascend to PAG and the posterolateral thalamus, for which the signals project to S1, S2, and ACC [21]; moreover, they could directly project through the midline and intralaminar thalamic nuclei to other limbic areas, including PAG, ACC, and amygdala [32]. Functionally, investigators reported that PAG demonstrated coherence with ACC (rostral and pregenual) in the resting state and formed a core intrinsic functional ACC-PAG-RVM network for pain modulation [31, 33]. Furthermore, Hui et al. summarized a series of their studies conducted over the last decade, and found that acupuncture analgesia was mainly relevant in terms of the deactivations in the limbic-paralimbic-neocortical network (LPNN), including PAG and ACC [16]. Consistent with these findings, our results may suggest that acupuncture therapy reverses the activation of limbic structures evoked by pain, resulting in an analgesic effect.

In 1968, Melzack and Casey described pain in terms of its three dimensions: “sensory-discriminative,” “affective-motivational,” and “cognitive-evaluative” [34]. Many studies have proved that the pACC and PAG are involved in not only acute pain but also emotion [31, 32]. The pACC has been found to be closely related to the affective network and could be specifically evoked by positive events [35], while PAG has been found to be significantly connected with its surrounding areas and is important for the control of emotions, for example, fear and the affective aspect of pain [31]. Furthermore, the aMCC and mammillary body may also be activated by negative emotion [36, 37]. Based on above analysis, we speculated that the unpleasantness of acute pain could induce dysfunction in these emotion-processing regions, and acupuncture may be beneficial for treating this dysfunction.

#### 4.2.2. Default Mode Network

The DMN generally shows specific spontaneous activations when a person is left undisturbed, for example, lying peacefully in an MRI or positron emission tomography scanner. Interestingly, these activations transform into coordinated deactivations during attention-demanding tasks such as pain or acupuncture stimuli [5, 6]. Anatomically, DMN comprises the regions along the anterior and posterior midline, the lateral parietal cortex, the prefrontal cortex, and the medial temporal lobe (MTL); it therefore overlaps with the limbic system to a certain degree. The precise function of DMN remains debatable; however, analysis of its intrinsic activity has revealed that its function might be divided into the MTL and dmPFC subsystems, with a midline core (PCC and anterior mPFC) [6].

Consistent with this finding, ACUP in the ALBP subjects yielded widespread deactivations in the PCC and MTL subsystems, including the HP/PHP, pMCC/PCN/PCC/retrosplenial cortex, and the angular gyrus, which play key roles in recalling the past or imagination of the future [6, 38]. In addition to DMN, the deactivated mammillary body, which usually acts as a relay for impulses coming from the amygdala and HP through the mamillothalamic tract to the thalamus, is part of the larger Papez circuit and is involved in storing memory [39]. Furthermore, investigators demonstrated that acupuncture could modulate memory encoding and retrieving in patients with mild cognitive impairment [40]. Consequently, we propose that acupuncture reduced spontaneous memory-related cognition, which might provide psychological relief from pain.

Besides the MTL subsystem, ACUP, also deactivated the dmPFC subsystem, including the supramarginal gyrus and the dmPFC. Prior studies have demonstrated that the dmPFC is linked with lower levels of autonomic outflow regions, including PAG and the hypothalamus in monkeys and rats, respectively [41, 42], and with the pACC in humans [43]. Clinical studies further found that acupuncture with the* de qi* sensation could inhibit the dmPFC for treating various psychological problems, such as schizophrenia and anxiety disorders [44, 45]. Moreover, acupuncture could decrease sympathetic activity and increase parasympathetic activity by inhibiting the dmPFC [46].

#### 4.2.3. Contact between the LPNN and DMN

Regulation of negative LPNN activity was a notable result of acupuncture. This network is thought to have significant relationships with pain conduction and changes in the brain function network involved with acupuncture regulation [16]; DMN of the brain overlaps with LPNN that is deactivated by acupuncture. Research has shown that, in terms of brain function, DMN interacted with LPNN, with broad activation. We confirmed Fang et al.'s conclusion [11] that this intrinsic organization may be a core function of LPNN network in response to ACUP.

### 4.3. Activation Network in Acupuncture Studies

Some researchers believe that ACUP serves as a somatosensory-guided mind-body therapy, which effectively combines peripheral sensory stimuli and cognitive ratings [10]. This view suggests that (1) active cognitive ratings during acupuncture evoked stronger* de qi* sensations than passive sensory stimuli [47]; (2) stronger de qi sensations evoked by acupuncture enhance more cognition than tactile stimulation [10]; and (3) paying attention to the pain can upregulate pain, while distraction can downregulate pain [48]. In addition, acupuncture stimuli could act as a placebo, and de qi sensation ratings may promote this placebo effect [49]. Broadly consistent with these views, our result in the ALBP subjects showed that both ACUP and SHAM, along with sensory stimuli and cognitive rating, evoke prominent activations in dlPFC and pMCC (Figures 7 and 8). Furthermore, the right insula and frontal operculum cortex activated by ACUP was another important attention-related area [50]. As previously observed, these four regions were thought to provide a higher level role in attentional control, including continuous monitoring of the external world, searching behavior for active solution derivation, and regulating the skeletomotor system in the presence of interfering stimuli [10, 32, 50–52]. Unlike the findings from a previous study [10], we found that ACUP evoked stronger* de qi* sensations in ALBP subjects, inducing weaker activations in both dlPFC and pMCC compared with SHAM. These differences can be explained by the fact that the* de qi* sensations were rated at end of each functional scan, rather than during each block. Moreover, previous researchers found that too little autonomic arousal may fail to activate the dlPFC, while too much attention focused upon a task may limit dlPFC function and selection of optimal responses in people with elevated anxiety [50]. Taken together, we considered that moderate activity in the attention network may be important for acupuncture analgesia.

In animal studies, researchers found that the analgesic effects of manual acupuncture may have mainly resulted from a C-type afferent, by means of selective blockade of conduction in C- and A*δ*-type afferents [4]. This effect seemed to act as a diffuse noxious inhibitory control, which also mediated C- and A*δ*-type afferents and strongly alleviated the initial painful sensation [53]. On the other hand, a fMRI study showed that moderate-high thermal pain on the right forearm activated the left sensorimotor regions (S1 and M1), bilateral insula, and S2, while deactivating the right sensorimotor regions (S1 and M1) [54]. However, a clinical study in carpal tunnel syndrome subjects showed that acupuncture in the right hand yielded significant deactivation in the left S1, supplementary motor area, and anterior insula compared with noninsertive cutaneous stimulation, although this was not found to be the case in healthy control subjects [55]. Our results in ALBP subjects showed that ACUP in the right leg deactivated the left sensorimotor regions (S2, M1, and insula) while activating the right sensorimotor regions (S1, M1, and insula). One reasonable explanation for our result is that the acupuncture stimuli may have inhibited the ipsilateral ascending nociceptive inputs and facilitated the contralateral inputs to some extent.

## 5. Limitation

This fMRI-based study of the analgesic mechanism of acupuncture provides a good foundation for future research. However, the study also has some limitations. First, the types of data differed, particularly, the baseline and the pain state data, were rs-fMRI type data, whereas the ACUP and SHAM data were task fMRI type data. Therefore, we could only compare the pain state data with the baseline in the ReHo model and compare the ACUP or SHAM status data with the OFF status data in the GLM model. We could not compare the rs-fMRI data with the task fMRI data. The data would be much better and more persuasive if they were of the same type and could be analyzed using the same method. Second, because each person's physique was different, the research subjects experienced different sensations of pain; it would be much better if we could divide the subjects into different groups according to their scores and for detailed analysis, similar to the* de qi* point. Third, although SHAM induced fewer deactivations than ACUP, it also yielded a somatic stimulatory effect on the ALBP model; to prove the effect of acupuncture, we could test other acupoints in the subjects and compare the results with the SHAM data.

## 6. Conclusion

In the present study, we found that ACUP induced more deactivations and fewer activations compared with SHAM. Furthermore, ACUP mainly induced deactivations in the limbic system and DMN, while mainly evoking activations in the attentional and somatosensory systems. Our results revealed that acupuncture analgesia may affect sensory, affective, cognitive, and autonomic functions, suggesting that acupuncture treatment exceeds somatosensory-guided mind-body therapy for ALBP. In addition, our experimental ALBP model may help to bridge the gap between clinical and experimental pain studies involving acupuncture treatment.

## Figures and Tables

**Table 1:** Resting state regional homogeneity alterations corresponding to pain status (pain status compared with baseline) paired *t*-test analysis *P* < 0.05, FDR < 0.05.

	BA	Cluster sizes	Peak *Z*-score	Peak MNI coordinate		
				*X*	*Y*	*Z*
R mPFC	8	32	3.02	20	27	60
R middle frontal gyrus	9	211	5.87	3	50	22
L middle temporal gyrus	21	54	−3.49	−70	−54	5
R superior temporal gyrus	38	32	−3.59	55	12	−30
L S1	2	59	−2.88	−60	−20	42
R inferior parietal lobule	40	20	−5.94	66	−36	20
L PHP	—	31	−2.62	−10	−3	−21
R PHP	35	29	4.51	30	−7	−21
L anterior cingulate cortex	32	42	−2.74	−6	25	39
R precuneus	7	54	2.70	19	−66	33
R insula	13	60	2.47	39	0	22
R cerebellar tonsil	—	53	2.42	11	−60	−48

FDR: false discovery rate; MNI: Montreal Neurological Institute; mPMC: medial prefrontal cortex; PHP: parahippocampus; S1: primary somatosensory cortex.

**Table 2:** fMRI signal changes evoked by ACUP (ACUP (ON status compared with OFF status)) one-sample *t*-test analysis *P* < 0.05, FDR < 0.05.

	BA	Cluster sizes	Peak *Z*-score	Peak MNI coordinate		
				*X*	*Y*	*Z*
Left insula	13	52	−3.51	−42	−15	15
Left M1	6	48	−2.56	−36	10	10
Left S2	43	36	−3.01	−37	−12	2
Left frontal eye field	8	66	−3.26	−15	35	53
Left dlPFC	46	97	3.81	−39	36	18
Right M1	6	348	6.16	51	6	12
Left PAG		44	−3.26	−3	−30	−3
Left PHP		35	−3.02	0	−24	0
Left thalamus		26	−2.56	4	−30	−5
Right dmPFC	8	43	−3.98	15	33	45
Right supramarginal gyrus	40	55	4.87	63	−27	33
Right S1	2	40	3.97	55	−20	30
Right supramarginal gyrus	40	48	−3.28	54	−60	39
Right angular gyrus	39	40	−3.00	50	−60	30
Right lateral temporal cortex	21	34	−2.56	49	−55	26
Right pMCC	31	101	3.98	18	−24	39
Bilateral SMA		79	2.54	12	0	60
Right PHP	35	46	−4.00	24	−27	−18
Right HP		40	−3.89	25	−20	−20
Bilateral pACC	32	34	−3.42	0	33	21
Left dmPFC	24	30	−3.23	15	20	20

FDR: false discovery rate; MNI: Montreal Neurological Institute; M1: primary motor cortex; S2: secondary somatosensory cortex; dlPFC: dorsolateral prefrontal cortex, periaqueductal grey (PAG); PHP: parahippocampus; dmPFC: dorsomedial prefrontal cortex; S1: primary somatosensory cortex; pMCC: posterior mid-cingulate cortex; SMA: supplementary motor area; HP: hippocampus; pACC: pregenual anterior cingulate cortex.

**Table 3:** fMRI signal changes evoked by SHAM (SHAM (ON status compared with OFF status)) one-sample *t*-test analysis *P* < 0.05, FDR < 0.05.

	BA	Cluster sizes	Peak *Z*-score	Peak MNI coordinate		
				*X*	*Y*	*Z*
Left insular	13	99	−3.60	−39	−12	15
Left frontal operculum	6	78	−3.46	−40	0	20
Left M1	44	60	−3.01	−23	−10	14
Left SMA	6	271	4.19	−27	3	57
Right dlPFC	46	80	4.01	−48	39	21
Right SMA	6	210	3.69	30	42	24
Left dlPFC	8	189	3.44	37	43	20
Right frontopolar area	9	154	3.23	30	25	30
Left mammillary body		456	5.67	−15	−3	3
Right thalamus		356	4.57	−12	0	0
Right mammillary body		234	3.58	19	23	8
Left thalamus		315	4.43	8	3	3
Amygdala		56	3.01	3	2	7
Left Hp	23	43	4.88	−27	−36	−6
Right PHP	36	32	3.45	−20	−23	0
Orbitofrontal cortex	37	34	3.03	−19	34	3
Right temporal pole	42	35	3.23	−24	−35	0
Left PCN	7	78	4.75	6	−57	36
Right pACC	23	56	3.89	5	−50	42
Left ACC	24	57	3.76	−4	−66	22
Left angular gyrus	19	49	3.35	27	−42	−27
Right angular gyrus	19	54	3.45	−28	−44	−34
Left cerebellum anterior lobe	—	46	3.25	34	−22	−20
Right cerebellum anterior lobe	—	45	3.24	−32	−10	20

FDR: false discovery rate; MNI: Montreal Neurological Institute; M1: primary motor cortex; SMA: supplementary motor area; dlPFC: dorsolateral prefrontal cortex; HP: hippocampus; PHP: parahippocampus, PCN: precuneus; pACC: pregenual anterior cingulate cortex; ACC: anterior cingulate cortex.

**Table 4:** fMRJ signal changes in the comparison of ACUP minus SHAM (ACUP compared with SHAM) paired *t*-test analysis *P* < 0.05, FDR < 0.05.

	BA	Cluster sizes	Peak *Z*-score	Peak MNI coordinate		
				*X*	*Y*	*Z*
Left SMA	6	97	−3.75	−24	12	60
Left frontal eye field	8	76	−3.45	−20	10	80
Right insular	13	160	3.79	39	9	9
Right M1		149	3.54	54	10	11
Right frontal eye field	8	54	−3.34	23	−10	70
Right dlPFC	38	42	−3.57	−54	12	−8
Left HP	24	234	−4.46	−12	−24	−6
Left PHP	23	121	−3.89	−10	−22	0
Left mammillary body	23	111	−3.65	−16	−19	22
Left thalamus		56	−2.79	12	34	0
Right PCC	21	76	−4.01	−3	−6	30
Left ACC		45	−2.58	4	5	−45
Right supramarginal gyrus	40	387	−4.34	57	−54	24
Right angular gyrus	22	134	−3.78	60	−34	20
Right precuneus	42	145	−3.89	45	−20	−30
Right thalamus	13	84	−4.09	24	−27	4
Right insular		76	−3.75	23	30	10
Right PHP	35	81	−4.06	24	−24	−18
Right HP	28	45	−3.06	30	−10	−29
Left dmPFC	24	99	−3.78	0	33	18
Left PAG	31	117	−4.31	−9	−48	37
Right cerebellar anterior lobe		234	−4.76	9	−60	40
Left cerebellar anterior lobe		320	−4.32	−18	56	33
Lateral occipital gyrus	10	87	−3.54	2	33	−18

FDR: false discovery rate; MNI: Montreal Neurological Institute; SMA: supplementary motor area; dlPFC: dorsolateral prefrontal cortex; HP: hippocampus; PHP: parahippocampus; pACC: pregenual anterior cingulate cortex; ACC: anterior cingulate cortex; dmPFC: dorsomedial prefrontal cortex.

## Experimental Site

All MRI scanning was conducted at the MRI Clinical Imaging Department at Zhujiang Hospital (Guangzhou, China).
